# Association of MiR-146a Expression and Type 2 Diabetes Mellitus: A Meta-Analysis

**DOI:** 10.22088/acadpub.BUMS.6.3.156

**Published:** 2017-08-14

**Authors:** Behnam Alipoor, Hamid Ghaedi, Reza Meshkani, Shahram Torkamandi, Sana Saffari, Mostafa Iranpour, Mir Davood Omrani

**Affiliations:** 1 *Department of Laboratory Sciences, Faculty of Paramedicine, Yasuj University of Medical Sciences, Yasuj, Iran.*; 2 *Department of Medical Genetics, Faculty of Medicine, Shahid Beheshti University of Medical Sciences, Tehran, Iran.*; 3 *Department of Biochemistry, Faculty of Medicine, Tehran University of Medical Sciences, Tehran, Iran.*; 4 *Department of Biology, Tehran North Branch, Islamic Azad University, Tehran, Iran.*; £ *Both authors contributed equally to this work.*

**Keywords:** Type 2 diabetes, microRNA-146a, meta-analysis

## Abstract

Although deregulation of miR-146a has been reported in type 2 diabetes repeatedly, the direction of deregulation events (up or down) remained to be inconsistent in literatures. Therefore, in this study we performed a meta-analysis on the possible association between miR-146a expression levels and type 2 diabetes. A systematic literature searching of PubMed, ISI Web of Science and Google Scholar was performed up to the end of September 2016. Finally, a total of 12 studies including 344 diabetic patients and 316 controls were selected for meta-analysis. All statistical analysis was performed using the metafor package with R software. Moreover, publication bias was assessed by Egger’s and sensitivity analysis was applied on the meta-analysis. The results are presented as log10 odds ratios (logORs), 95% confidence intervals (CI) with relevant P values. The results revealed that miR-146a was downregulated in type 2 diabetes cases compared with normal subjects (P=0.01, logOR:-4.76, 95% CI:-8.41, -1.11). Furthermore, sub-group analysis showed that the association between miR-146a expression levels and type 2 diabetes in whole blood (P<0.001) and PBMCs (P<0.001) samples were significant. However, this association was not significant in the serum (P=0.67) and plasma (P=0.90) samples. Our finding suggests that miR-146a downregulation could be associated with type 2 diabetes susceptibility. Further investigations with larger sample size are required to evaluate this association in the type 2 diabetes pathogenesis.

Type 2 diabetes (T2D) is the most common form of diabetes resulting from interactionof genetic and environmental factors (1). This metabolic disorder is characterized by hypergl-ycemia, insulin resistance and finally pancreatic β-cell dysfunction (2). Epidemiologic studies have shown that in 2013, 382 million people had diabetes and predicted and this number is expected to rise to 592 million by 2035 (3). T2D is associated with several complications such as cardiovascular disorders, retinopathy and nephropathy which can reduce the quality of life and life expectancy. Therefore, exploring novel early biomarkers that identify individuals susceptible to develop severe complications could help clinicians to choose the best therapeutic approaches (4, 5).

Since the discovery in 1993, there has been a steady increase in the number of studies investigating the role of circulatory microRNAs (miRNAs) as highly stable and non-invasive biomarkers (6, 7). MiRNAs are short 20–23 nucleotides non-coding RNAs molecules that modulate gene expression at post-transcriptional level by binding to 3' UTR target sites of mRNAs (8). They have essential roles in homeostasis of glucose and lipid metabolism, development of pancreatic β cells, production and secretion of insulin (9, 11). The increasing number of studies suggested that deregulation of miRNAs may occur in individuals susceptible to T2D. Besides, it has been shown that altered miRNAs expression levels were associated with early detection, clinical outcomes and severity of complications in different pathological situations including T2D (12-14).

To date, several studies have provided important evidence about the role of circulating miRNAs as biomarkers for T2D (5-14). The miRNA-146a (miR-146a) is one of the most important miRNAs that its deregulation has been implicated in T2D pathogenesis. Although upregulation/ deregulation of miR-146a expression level has been reported in T2D repeatedly, the direction of deregulation events (up or down) remained to be inconsistent in literatures. There is much evidence showing that the expression levels of miR-146a significantly decreased in the peripheral blood mononuclear cells (PBMCs), plasma and serum samples of patients with T2D compared with control subjects (15-18, 23). Nonetheless, in contrast to these results, it has been reported that miR-146a serum level was significantly upregulated in newly diagnosed T2D patients compared to individuals with normal glucose tolerance (22). The inconsistency may be due to the use of small sample size, different profiling methods and sample sources. Hence, to obtain a robust comprehension, here we aimed at pooling studies of miR-146a expression in T2D samples compared with healthy controls, and then performing a meta-analysis while considering different confounding variables.

## Methods

**Search strategy**

A Systematic literature search using PubMed, ISI Web of Science and Google Scholar were performed for relevant articles from 1993 to September 2016 and the language was restricted to English. To find eligible articles, a combination of the following key words was used: “miR-146a”, “expression”, “profiling”, “type 2 diabetes”, “circulating”, “plasma”, “serum”, and “blood”. In additon, to identify additional eligible studies, we scanned the reference lists of reviews and publications selected for inclusion in this paper.

**Selection criteria and publication quality assessment**

Studies were eligible if they met the following criteria: (1) had miRNAs expression profiling design, (2) compared patients with diabetes and healthy controls, (3) with information on cut-off criteria (P-value or fold-change) for differentially expressed genes, (4) studied human circulatory miRNAs (whole blood, PBMC, serum, plasma and platelet), and (5) reported total number of samples in both patient and control groups. We excluded review articles and studies that determined miRNAs expression profiling on human tissues.

**Data extraction and quality assessment**

The required data were retrieved by two independent investigators from the full- text eligible articles and supplementary materials. Any disagreement was resolved by a team discussion. For each study, the following information and characteristics were collected and recorded: the first author’s name, year of publication, paper DOI number, study population, miRNAs expression profiling technique, number of cases and controls, number of studied miRNAs, type of sample, cut-off value and type of dysregulation (upregulation or downregulation). With regardto the technique used in a given study, microarray platform or qPCR, quality assessment of data was performed according to the Minimum Information About a Microarray Experiment (MIAME) guidelines version 2 and Minimum Information for Publication of Quantitative Real-time PCR Experiments (MIQE).

**Statistical analysis**

All analyses were performed using the metafor package with R statistical software 3.1. Meta-analysis was performed as described elsewhere (24). To assess the statistical significance, we made 2x2 contingency table for miR-146a based on the number of dysregulation events in both type 2 diabetes and non-diabetic control subjects. The outcomes are presented as log10 odds ratios (logORs), with their 95% confidence intervals. The miRNAs upregulation was indicated when a significant logOR higher than one was obtained from cases to controls. on contrary, miRNAs downregulation was indicated with a significant logOR higher than one, whenever controls were compared to cases. The significance of the pooled logOR was determined by the Z-test and P<0.05 was considered as statistically significant. We quantified the possibility of heterogeneity between studies and the proportion of inter-study variability by Cochran's Q-statistic and I^2^ statistics, respectively. If a difference in statistical heterogeneity was detected (PQ<0.10 or I^2^≥50%), a random-effects model (fitted by restricted maximum-likelihood estimator) was applied to the pooled logORs. Otherwise, the ﬁxed-effects model was used. To assess the stability of acquired P-values, leave-one-out (sensitivity analysis) was applied on the meta-analysis. Additionally, since the sample sources (whole blood, serum, plasma and PBMC) were different, a sub-group analysis was performed. Also, publication bias was examined by a funnel plot and funnel plot asymmetry was assessed by Egger’s linear regression test. The significance of the intercept was determined by the t test, as proposed by Egger, with P<0.10 considered indicative of statistically significant publication bias. Otherwise, the study was considered to have no publication bias.

## Results

**The characteristics of included studies**

After titles and abstracts initial screening, 10 related articles were obtained by literature search from PubMed, ISI Web of Science and Google Scholar databases. Further, we added our work as unpublished study (consisted of two sub-studies). Finally, a total of 12 studies/sub-studies including 344 T2D patients and 316 normal samples were selected for meta-analysis (Table 1).

**Meta-analysis of miR-146a expression in T2D**

**Fig. 1:**
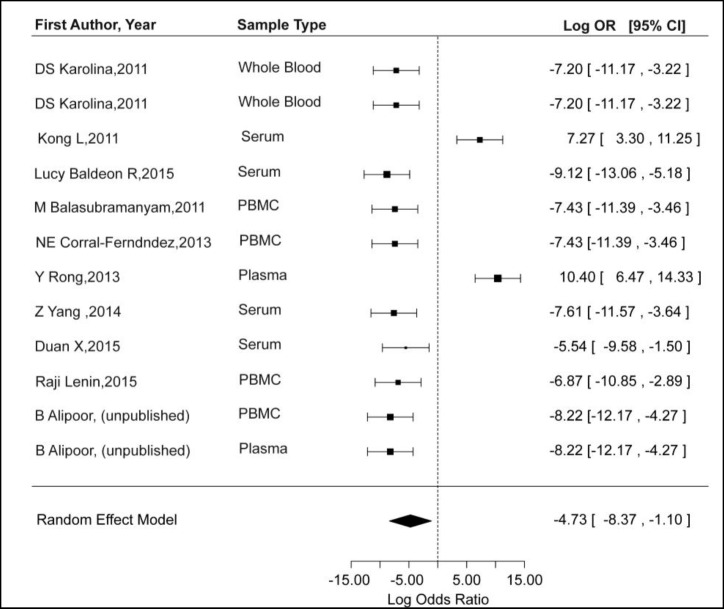
Forest plot analysis for the association of miR-146a expression level and T2D.

**Fig. 2:**
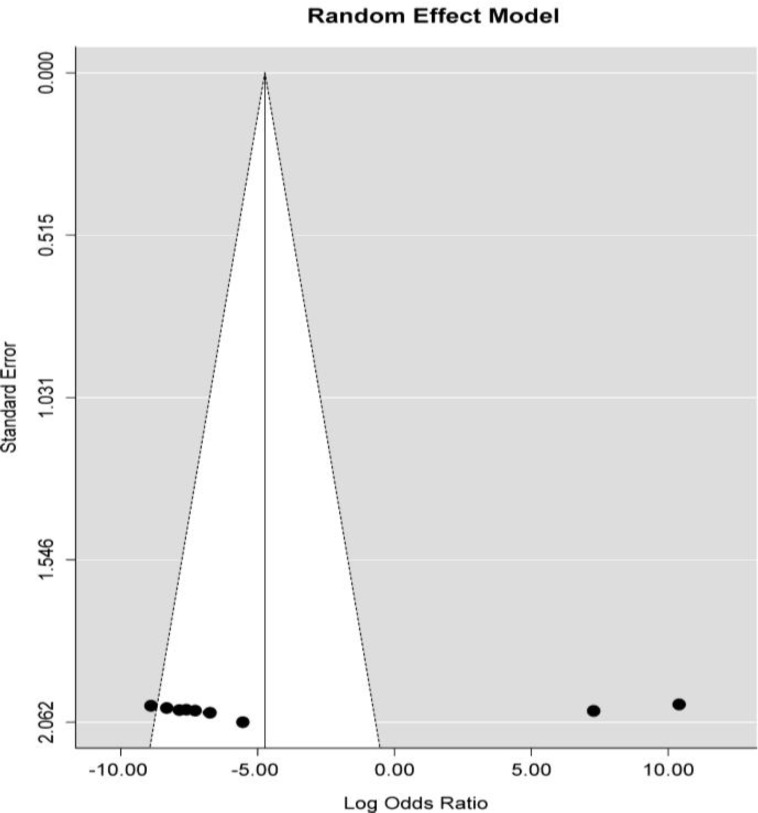
Funnel plot of publication bias**. **Egger’s test was applied to assess funnel asymmetry and the statistics was t= -0.67, df= 10, P value=0.51.

We applied a random-effects model for the meta-analysis, since I^2^ (total heterogeneity/ total variability) was as 90.15%. Overall, meta-analysis revealed that miR-14a was downregulated in T2D cases compared with controls (P= 0.01, logOR: -4.76, 95% CI: -8.41, -1.11) (Figure 1). As a further matter, the publication bias was assessed by a funnel plot. As demonstrated by the funnel plot, there was no significant publication bias in meta-analysis (P for Egger’s test<0.10) (Figure 2). Subsequently, sensitivity analyses were performed to evaluate the effect of each individual study on the pooled ORs by sequential omitting of studies (Table 2). Indeed, we found that Kong et al. (22) and Rong et al. (23) studies influence the pooled effect. Since these studies have shown miR-146a upregulation in T2D patients, leaving them out of analysis made the pooled effect to become more statistically significant.

Finally, to evaluate the sample type effects on the meta-analysis, we performed sub-group analysis (Table 3). Our analysis revealed that the association between miR-146a expression levels and T2D in whole blood (P<0.001) and PBMC (P<0.001) samples were significant. Nevertheless, we did not observe a significant association when we evaluated this correlation in the serum (P=0.67) and plasma (P=0.90) samples.

**Table 1:** Characteristics of studies included in the meta-analysis

First Author (reference)	Year	Sample	T2D (n)	Control s(n)	Method	U/D^1^	P
Karolina DS ^(15)^	2011	Whole blood	21	15	qPCR	D	0.001
Karolina DS ^(15)^	2011	Whole blood	21	15	microarray	D	0.05
Duan X ^(16)^	2015	Serum	7	8	qPCR	D	0.007
Kong L ^(22)^	2011	Serum	18	19	qPCR	U	0.008
Baldeón L ^(17)^	2015	PBMC	48	34	qPCR	D	0.05
Balasubramanyam M ^(18)^	2011	PBMC	20	20	qPCR	D	0.001
Corral-FernándezN ^(19)^	2013	PBMC	20	20	qPCR	D	0.01
Rong Y ^(23)^	2013	Plasma	90	90	qPCR	U	0.05
Yang Z ^(20)^	2014	Serum	24	20	qPCR	D	0.102
Lenin R ^(21)^	2015	PBMC	15	15	qPCR	D	0.001
Alipoor B ^(2)^	2016	PBMC	30	30	qPCR	D	0.004
Alipoor B ^(2)^	2016	Plasma	30	30	qPCR	D	0.008

1: U stands for up-regulation and D stands for down-regulation; 2: unpublished study.

**Table 2:** Sensitivity analysis using leave-one-out method

**First Author, Sample type**	**LogOR [95% CI]**	**Z test**	**P value**	**I** ^2^
DS Karolina, Whole blood	-4.54 [-8.50,-0.57]	-2.24	0.02	90.85
DS Karolina, Whole blood	-4.54 [-8.50,-0.57]	-2.234	0.02	90.85
Kong L, Serum	-5.85 [-9.09,-2.61]	-3.54	0.00	86.25
Lucy Baldeon R, Serum	-4.36 [-8.27,-0.45]	-2.18	0.02	90.60
M Balasubramanyam, PBMC	-4.51 [-8.48,-0.55]	-2.23	0.02	90.83
Corral-Ferndndez, PBMC	-4.51 [-8.48,-0.55]	-2.23	0.02	90.83
Y Rong, Plasma	-6.14 [-8.82,-3.45]	-4.48	0.00	79.91
Z Yang , Serum	-4.50 [-8.46,-0.54]	-2.22	0.02	90.80
Duan X, Serum	-4.69 [-8.68,-0.69]	-2.30	0.02	91.00
Raji Lenin, PBMC	-4.56 [-8.54,-0.59]	-2.24	0.02	90.89
B Alipoor, PBMC	-4.44 [-8.38,-0.50]	-2.21	0.02	90.71
B Alipoor, Plasma	-4.44[-8.38,-0.50]	-2.21	0.02	90.71

**Table 3:** Sub-set analysis by sample types

**Sample type**	**Test of significance**		
	**LogOR [95%CI]**	**Z test**	**P value**
Whole blood	-7.19 [-10.00, -4.38]	-5.01	<0.00
Serum	-1.95 [-11.08, 7.17 ]	-0.41	0.67
PBMCs	-7.75 [-9.52,-5.98]	-8.5	<0.00
Plasma	1.08 [-17.15,19.33]	0.11	0.90

## Discussion

T2D is a silent slow progressive killer disease resulting from chronic metabolic disorder in the body which is characterized by hyperglycemia, insulin resistance and finally pancreatic β-cell dysfunction (2). Consequently, early diagnosis is the key point for diabetic patients to take appropriate therapeutic treatment (2, 25). There is a growing body of evidence suggesting the role of circulatory miRNAs as novel biomarkers for early diagnosis of T2D (5, 14). Although there are several reports showing that a large number of circulating miRNAs were deregulated in T2D, dis-agreements still exist to determine which miRNAs are upregulated or downregulated. The inconsis-tency may be due to the use of small sample size, different profiling methods and sample sources. As a consequence, in this study we performed a meta-analysis on the possible association between miR-146a expression levels and T2D.

Our results revealed that miR-146a was downregulated in T2D samples compared with controls. The miR-146a is one of the most important miRNAs that its deregulation has been implicated in diabetes. Accumulating evidence has reported that miR-146a regulates the genes involved in the pathogenesis of T2D and its related complications. It has been suggested that chronic inflammation is an important determinant in insulin resistance and also microvascular complications of T2D including nephropathy, neuropathy and retinopathy (4). MiR-146a is involved in the regulation of toll like receptors (TLRs) signaling pathway in innate immune system. so, the reduction in miR-146a expression could lead to less efficient inhibition of target genes involved in the TLRs and other cytokine production and signaling pathways. Studies have shown that miR-146a interacts directly with interleukin-1 receptor-associated kinase 1 (*IRAK1*)/TNF receptor-associated factor 6 (*TRAF6)*, thus attenuating the inflammatory cytokines production in macrophages (26, 27). It has been reported that patients suffering from T2D have significantly decreased levels of miR-146a (15, 21). yet, in contrast to these ﬁndings, an increased expression of this miRNA has also been previously reported (5, 14).

Further, the subgroup analysis by sample type showed that the association between miR-146a expression level and T2D was significant in PBMCs and whole blood samples, whereas it was not significant for serum and plasma samples. As a result, determination of miR-146a expression level as molecular marker for T2D can be more useful in PBMCs than in serum and plasma. One possible reason for this finding may be due to the fact that miRNAs expressions are cell and tissue specific (28). It has been shown that miR-146a levels are much more abundant in the PBMCs including lymphocytes and macrophages/monocytes (29).

Importantly, there is a report which performed a meta-analysis on miR-146a expression in different tissues in human and model organisms (24). Although we used similar methodology to conduct meta-analysis, our study differs in several aspects regarding this study. First, we considered only miR-146a which is presented in circulatory sources (serum, plasma and PBMCs). Second we only included studies with human subjects. Considering such criteria makes our study more accurate in determining the potential role of miR-146a as non-invasive biomarker for T2D and its complications.

Even though we tried to perform a well-designed and robust meta-analysis, the present study may suffer from some limitations. First, the existing bias in publishing of reports has significant result. Second, in the subgroup analysis using sample types, the number of studies in each group was relatively small. Third, methodological limitation in miRNAs expression meta-analysis analyzed all studies with non-significant results. Four, because our meta-analysis did not estimate the contribution of other risk factors related to T2D and also the stage of disease, these variables may have influenced the result of our study and partially explain the discrepancies in the involved case-control studies.

In conclusion, our finding suggest that miR-146a downregulation could be associated with T2D susceptibility. Furthermore, this result suggests that miR-146a can be considered as a potent marker to predict the clinical outcome of diabetes. This association needs to be confirmed through more clinical investigations with larger sample size and well characterized study populations.
